# 4′-Hydroxy­biphenyl-4-carboxylic acid

**DOI:** 10.1107/S1600536808014220

**Published:** 2008-05-17

**Authors:** Sun Feng

**Affiliations:** a School of Chemistry and Environment, South China Normal University, Guangzhou 510006, People’s Republic of China

## Abstract

The title compound, C_13_H_10_O_3_, has potential oxygen donor and acceptor sites. Inter­molecular hydrogen bonding between neighboring carboxyl­ate groups leads to the formation of hydrogen-bonded dimers [graph-set motif *R*_2_^2^(8)]. A second hydrogen-bonding inter­action between the hydr­oxy groups generates a chain and extends the structure into a lamellar layer. One of the benzene rings is disordered over two positions with an occupancy ratio of 0.57 (2):0.43 (2).

## Related literature

For related literature, see: Bernstein *et al.* (1995 ▶); Datta & Pati (2006 ▶); Zwier *et al.* (1996 ▶).
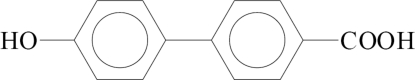


## Experimental

### 

#### Crystal data

C_13_H_10_O_3_
*M* *_r_* = 214.21
Monoclinic, 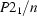

*a* = 8.6500 (7) Å
*b* = 5.5077 (5) Å
*c* = 20.9655 (18) Å
β = 94.145 (3)°
*V* = 996.22 (15) Å^3^
*Z* = 4
Mo *K*α radiation
μ = 0.10 mm^−1^
*T* = 293 (2) K
0.21 × 0.20 × 0.16 mm

#### Data collection

Bruker APEXII area-detector diffractometer
Absorption correction: none
6310 measured reflections
1800 independent reflections
854 reflections with *I* > 2σ(*I*)
*R*_int_ = 0.063

#### Refinement

*R*[*F* ^2^ > 2σ(*F* ^2^)] = 0.058
*wR*(*F* ^2^) = 0.183
*S* = 1.01
1800 reflections
160 parameters
24 restraints
H-atom parameters constrained
Δρ_max_ = 0.18 e Å^−3^
Δρ_min_ = −0.19 e Å^−3^

### 

Data collection: *APEX2* (Bruker, 2004 ▶); cell refinement: *APEX2*; data reduction: *APEX2*; program(s) used to solve structure: *SHELXS97* (Sheldrick, 2008 ▶); program(s) used to refine structure: *SHELXL97* (Sheldrick, 2008 ▶); molecular graphics: *XP* in *SHELXTL* (Sheldrick, 2008 ▶); software used to prepare material for publication: *SHELXTL*.

## Supplementary Material

Crystal structure: contains datablocks I, global. DOI: 10.1107/S1600536808014220/zl2111sup1.cif

Structure factors: contains datablocks I. DOI: 10.1107/S1600536808014220/zl2111Isup2.hkl

Additional supplementary materials:  crystallographic information; 3D view; checkCIF report

## Figures and Tables

**Table 1:** Hydrogen-bond geometry (Å, °)

*D*—H⋯*A*	*D*—H	H⋯*A*	*D*⋯*A*	*D*—H⋯*A*
O2—H2⋯O1^i^	0.82	1.82	2.624 (3)	168
O3—H3*A*⋯O3^ii^	0.82	2.20	3.0041 (18)	168

Symmetry codes: (i) 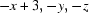; (ii) 
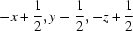
.

**Fig. 1.**
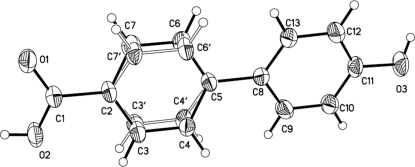
The structure of (I), showing the atom-numbering scheme and displacement ellipsoids drawn at the 30% probability level.

**Fig. 2.**
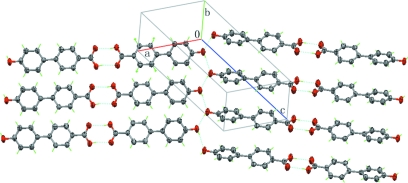
A layer view of (I).

## supplementary crystallographic information

### Comment

Hydrogen-bonding interactions between ligands are specific and directional. When present in metal complexes they usually do not rely on the properties of the metal ions, but they play an important role in the overall structures and functions of the complexes and the way in which they pack in the solid state (Zwier *et al.*, 1996; Datta & Pati, 2006). In this context we report here the crystal structure of the title compound, (I).

The molecular structure of (I) is shown in Fig. 1. The C—O and C—C bond distances show no remarkable features. The title molecular structure acts as both a hydrogen bonding donor and acceptor, forming dimers with neighboring molecules through O—H···O hydrogen bonding with a *R*^2^_2_(8) graph set motif (Bernstein *et al.*, 1995). A second hydrogen bonding interaction by the hydroxyl groups forms a chain and extends the structure into a lamellar layer (Table 1, Fig. 2).

### Experimental

4-Hydroxyl-biphenyl-4'-carboxylic acid was dissolved in a hot ethanol-water solution (1:1; *v*/*v*) with stirring. Colorless single crystals suitable for X-ray diffraction were obtained at room temperature by slow evaporation of the solvent over a period of several days.

### Refinement

In the initial refinement with disorder not taken into account one of phenyl rings showed significantly elongated thermal ellipsoids indicating disorder, the dihedral angle between two phenyl rings is 5.66 (2) /%A, and the adjacent distances of C-H···C-H interactions in the biphenylene are 2.044 (1) and 2.077 (1) /%A, respectively, thus leading to a static repulsion between two phenyl rings,and the phenyl ring was thus refined as being disordered over two positions. The occupancy ratio refined to 0.57 (2) to 0.43 (2). The adps of the disordered atoms were restrained to be close to isotropic and those of equivalent atoms were set to be identical. Carbon-bound, hydroxyl and carboxylate group H atoms were placed at calculated positions and were treated as riding on their parent C or O atoms with C—H = 0.93 Å, with *U*_iso_(H) = 1.2 *U*_eq_(C); O—H = 0.82 Å and with *U*_iso_(H) = 1.5 *U*_eq_(O).

### Crystal data

**Table:**

C_13_H_10_O_3_	*F*_000_ = 448
*M**_r_* = 214.21	*D*_x_ = 1.428 Mg m^−^^3^
Monoclinic, *P*2_1_/*n*	Mo *K*α radiation λ = 0.71073 Å
Hall symbol: -P 2yn	Cell parameters from 1560 reflections
*a* = 8.6500 (7) Å	θ = 1.4–28.0º
*b* = 5.5077 (5) Å	µ = 0.10 mm^−^^1^
*c* = 20.9655 (18) Å	*T* = 293 (2) K
β = 94.145 (3)º	Plate, colorless
*V* = 996.22 (15) Å^3^	0.21 × 0.20 × 0.16 mm
*Z* = 4	

### Data collection

**Table:**

Bruker APEXII area-detector diffractometer	854 reflections with *I* > 2σ(*I*)
Radiation source: fine-focus sealed tube	*R*_int_ = 0.063
Monochromator: graphite	θ_max_ = 25.2º
*T* = 293(2) K	θ_min_ = 2.0º
φ and ω scans	*h* = −10→10
Absorption correction: none	*k* = −5→6
6310 measured reflections	*l* = −24→25
1800 independent reflections	

### Refinement

**Table:**

Refinement on *F*^2^	Secondary atom site location: difference Fourier map
Least-squares matrix: full	Hydrogen site location: inferred from neighbouring sites
*R*[*F*^2^ > 2σ(*F*^2^)] = 0.058	H-atom parameters constrained
*wR*(*F*^2^) = 0.183	*w* = 1/[σ^2^(*F*_o_^2^) + (0.0772*P*)^2^] where *P* = (*F*_o_^2^ + 2*F*_c_^2^)/3
*S* = 1.01	(Δ/σ)_max_ < 0.001
1800 reflections	Δρ_max_ = 0.18 e Å^−^^3^
160 parameters	Δρ_min_ = −0.19 e Å^−^^3^
24 restraints	Extinction correction: none
Primary atom site location: structure-invariant direct methods	

### Special details

**Table:**

Geometry. All e.s.d.'s (except the e.s.d. in the dihedral angle between two l.s. planes) are estimated using the full covariance matrix. The cell e.s.d.'s are taken into account individually in the estimation of e.s.d.'s in distances, angles and torsion angles; correlations between e.s.d.'s in cell parameters are only used when they are defined by crystal symmetry. An approximate (isotropic) treatment of cell e.s.d.'s is used for estimating e.s.d.'s involving l.s. planes.
Refinement. Refinement of *F*^2^ against ALL reflections. The weighted *R*-factor *wR* and goodness of fit *S* are based on *F*^2^, conventional *R*-factors *R* are based on *F*, with *F* set to zero for negative *F*^2^. The threshold expression of *F*^2^ > σ(*F*^2^) is used only for calculating *R*-factors(gt) *etc*. and is not relevant to the choice of reflections for refinement. *R*-factors based on *F*^2^ are statistically about twice as large as those based on *F*, and *R*- factors based on ALL data will be even larger.

### Fractional atomic coordinates and isotropic or equivalent isotropic displacement parameters (Å^2^)

**Table:**

	*x*	*y*	*z*	*U*_iso_*/*U*_eq_	Occ. (<1)
C1	1.3074 (3)	0.0387 (7)	0.03781 (15)	0.0499 (9)	
C8	0.7212 (3)	0.1508 (6)	0.15024 (14)	0.0448 (8)	
C9	0.6208 (3)	0.3440 (6)	0.13452 (16)	0.0578 (10)	
H9	0.6499	0.4597	0.1054	0.069*	
C10	0.4806 (3)	0.3688 (7)	0.16071 (17)	0.0593 (10)	
H10	0.4167	0.4999	0.1493	0.071*	
C11	0.4352 (3)	0.2019 (7)	0.20337 (15)	0.0525 (9)	
C12	0.5302 (4)	0.0092 (7)	0.22074 (16)	0.0596 (10)	
H12	0.5001	−0.1052	0.2500	0.072*	
C13	0.6703 (4)	−0.0119 (6)	0.19414 (15)	0.0545 (9)	
H13	0.7339	−0.1424	0.2064	0.065*	
O1	1.3852 (2)	−0.1522 (5)	0.05016 (11)	0.0704 (8)	
O2	1.3483 (2)	0.2037 (5)	0.00069 (12)	0.0696 (8)	
H2	1.4342	0.1717	−0.0112	0.104*	
O3	0.2936 (3)	0.2362 (5)	0.22900 (12)	0.0720 (8)	
H3A	0.2672	0.1099	0.2458	0.108*	
C2	1.15866 (19)	0.0702 (5)	0.06838 (10)	0.0480 (8)	0.43 (2)
C3	1.0867 (8)	0.2959 (6)	0.0682 (6)	0.052 (3)	0.43 (2)
H3	1.1327	0.4288	0.0498	0.063*	0.43 (2)
C4	0.9458 (8)	0.3228 (6)	0.0954 (6)	0.045 (2)	0.43 (2)
H4	0.8976	0.4738	0.0952	0.054*	0.43 (2)
C5	0.8770 (2)	0.1241 (4)	0.12279 (11)	0.0441 (8)	0.43 (2)
C6	0.9490 (7)	−0.1015 (7)	0.1230 (5)	0.045 (3)	0.43 (2)
H6	0.9029	−0.2345	0.1413	0.054*	0.43 (2)
C7	1.0898 (8)	−0.1285 (7)	0.0958 (6)	0.052 (3)	0.43 (2)
H7	1.1380	−0.2795	0.0959	0.062*	0.43 (2)
C2'	1.15879 (19)	0.0709 (5)	0.06822 (10)	0.0480 (8)	0.57 (2)
C3'	1.0571 (7)	0.2579 (15)	0.0469 (5)	0.052 (2)	0.57 (2)
H3'	1.0845	0.3647	0.0153	0.063*	0.57 (2)
C4'	0.9160 (7)	0.2818 (15)	0.0734 (5)	0.049 (2)	0.57 (2)
H4'	0.8485	0.4045	0.0588	0.059*	0.57 (2)
C5'	0.8725 (2)	0.1234 (5)	0.12205 (11)	0.0441 (8)	0.57 (2)
C6'	0.9755 (6)	−0.0581 (16)	0.1431 (4)	0.048 (2)	0.57 (2)
H6'	0.9505	−0.1621	0.1758	0.057*	0.57 (2)
C7'	1.1161 (6)	−0.0845 (15)	0.1155 (4)	0.047 (2)	0.57 (2)
H7'	1.1827	−0.2097	0.1291	0.056*	0.57 (2)

### Atomic displacement parameters (Å^2^)

**Table:**

	*U*^11^	*U*^22^	*U*^33^	*U*^12^	*U*^13^	*U*^23^
C1	0.0396 (18)	0.061 (2)	0.051 (2)	0.0010 (18)	0.0134 (16)	0.0005 (19)
C8	0.0388 (17)	0.044 (2)	0.0527 (18)	−0.0001 (16)	0.0113 (15)	−0.0022 (17)
C9	0.0445 (19)	0.058 (2)	0.073 (2)	0.0005 (18)	0.0159 (17)	0.011 (2)
C10	0.0418 (19)	0.057 (2)	0.081 (2)	0.0079 (18)	0.0170 (18)	0.008 (2)
C11	0.0338 (17)	0.062 (3)	0.064 (2)	−0.0032 (17)	0.0160 (15)	−0.0135 (19)
C12	0.049 (2)	0.061 (3)	0.071 (2)	0.0015 (18)	0.0214 (18)	0.0075 (19)
C13	0.0448 (19)	0.051 (2)	0.069 (2)	0.0088 (17)	0.0183 (17)	0.0053 (18)
O1	0.0529 (15)	0.0705 (18)	0.0910 (18)	0.0171 (13)	0.0281 (13)	0.0188 (15)
O2	0.0460 (14)	0.083 (2)	0.0837 (18)	0.0127 (13)	0.0307 (13)	0.0194 (15)
O3	0.0418 (13)	0.090 (2)	0.0882 (18)	0.0033 (13)	0.0308 (12)	−0.0057 (16)
C2	0.0334 (17)	0.062 (2)	0.050 (2)	−0.0008 (17)	0.0144 (15)	0.0031 (18)
C3	0.040 (5)	0.065 (7)	0.054 (5)	0.002 (5)	0.015 (4)	0.006 (5)
C4	0.046 (5)	0.047 (6)	0.043 (5)	0.005 (4)	0.012 (4)	0.000 (4)
C5	0.0367 (17)	0.049 (2)	0.0476 (19)	−0.0041 (16)	0.0129 (14)	0.0004 (17)
C6	0.045 (5)	0.052 (5)	0.039 (5)	−0.002 (4)	0.002 (4)	−0.008 (4)
C7	0.041 (5)	0.061 (7)	0.052 (5)	0.003 (4)	0.001 (4)	−0.001 (5)
C2'	0.0334 (17)	0.062 (2)	0.050 (2)	−0.0008 (17)	0.0144 (15)	0.0031 (18)
C3'	0.044 (4)	0.057 (5)	0.058 (4)	0.004 (3)	0.014 (4)	0.008 (4)
C4'	0.042 (4)	0.049 (4)	0.057 (4)	0.012 (3)	0.014 (3)	0.000 (4)
C5'	0.0367 (17)	0.049 (2)	0.0476 (19)	−0.0041 (16)	0.0129 (14)	0.0004 (17)
C6'	0.036 (4)	0.068 (5)	0.039 (4)	0.007 (3)	0.010 (3)	0.009 (4)
C7'	0.029 (3)	0.063 (5)	0.049 (4)	0.011 (3)	0.005 (3)	0.007 (4)

### Geometric parameters (Å, °)

**Table:**

C1—O2	1.264 (4)	C3—C4	1.3900
C1—O1	1.265 (4)	C3—H3	0.9300
C1—C2	1.488 (4)	C4—C5	1.3900
C8—C13	1.379 (4)	C4—H4	0.9300
C8—C9	1.398 (4)	C5—C6	1.3900
C8—C5	1.510 (3)	C6—C7	1.3900
C9—C10	1.374 (4)	C6—H6	0.9300
C9—H9	0.9300	C7—H7	0.9300
C10—C11	1.360 (5)	C2'—C7'	1.3793
C10—H10	0.9300	C2'—C3'	1.4061
C11—C12	1.375 (4)	C3'—C4'	1.3820
C11—O3	1.386 (4)	C3'—H3'	0.9300
C12—C13	1.375 (4)	C4'—C5'	1.4147
C12—H12	0.9300	C4'—H4'	0.9300
C13—H13	0.9300	C5'—C6'	1.3903
O2—H2	0.8200	C6'—C7'	1.3923
O3—H3A	0.8200	C6'—H6'	0.9300
C2—C3	1.3900	C7'—H7'	0.9300
C2—C7	1.3900		
			
O2—C1—O1	123.7 (3)	C3—C4—C5	120.0
O2—C1—C2	118.1 (3)	C3—C4—H4	120.0
O1—C1—C2	118.2 (3)	C5—C4—H4	120.0
C13—C8—C9	115.4 (3)	C6—C5—C4	120.0
C13—C8—C5	121.9 (3)	C6—C5—C8	119.8 (2)
C9—C8—C5	122.7 (3)	C4—C5—C8	120.1 (2)
C10—C9—C8	122.2 (3)	C5—C6—C7	120.0
C10—C9—H9	118.9	C5—C6—H6	120.0
C8—C9—H9	118.9	C7—C6—H6	120.0
C11—C10—C9	120.1 (3)	C6—C7—C2	120.0
C11—C10—H10	120.0	C6—C7—H7	120.0
C9—C10—H10	120.0	C2—C7—H7	120.0
C10—C11—C12	120.1 (3)	C7'—C2'—C3'	119.2
C10—C11—O3	117.9 (3)	C4'—C3'—C2'	119.5
C12—C11—O3	122.0 (3)	C4'—C3'—H3'	120.2
C11—C12—C13	119.0 (3)	C2'—C3'—H3'	120.2
C11—C12—H12	120.5	C3'—C4'—C5'	121.3
C13—C12—H12	120.5	C3'—C4'—H4'	119.4
C12—C13—C8	123.3 (3)	C5'—C4'—H4'	119.4
C12—C13—H13	118.4	C6'—C5'—C4'	118.4
C8—C13—H13	118.4	C5'—C6'—C7'	120.1
C3—C2—C7	120.0	C5'—C6'—H6'	120.0
C3—C2—C1	120.3 (2)	C7'—C6'—H6'	120.0
C7—C2—C1	119.7 (2)	C2'—C7'—C6'	121.5
C4—C3—C2	120.0	C2'—C7'—H7'	119.3
C4—C3—H3	120.0	C6'—C7'—H7'	119.3
C2—C3—H3	120.0		

### Hydrogen-bond geometry (Å, °)

**Table:**

*D*—H···*A*	*D*—H	H···*A*	*D*···*A*	*D*—H···*A*
O2—H2···O1^i^	0.82	1.82	2.624 (3)	168
O3—H3A···O3^ii^	0.82	2.20	3.0041 (18)	168

Symmetry codes: (i) −*x*+3, −*y*, −*z*; (ii) −*x*+1/2, *y*−1/2, −*z*+1/2.
